# Rationale and design to assess the efficacy and safety of HT047 in patients with acute ischemic stroke: A multicenter, randomized, double-blind, placebo-controlled, parallel-group, phase II trial

**DOI:** 10.1097/MD.0000000000017655

**Published:** 2019-10-25

**Authors:** Sung Hyuk Heo, Jungbin Song, Bum Joon Kim, Hocheol Kim, Dae-Il Chang

**Affiliations:** a Department of Neurology, Kyung Hee University Hospital; b Department of Herbal Pharmacology, Kyung Hee University College of Korean Medicine, Seoul, Republic of Korea.

**Keywords:** clinical trial, HT047, ischemic stroke, motor function, *Pueraria lobata*, *Scutellaria baicalensis*

## Abstract

**Background:**

Though several neuroprotective agents have been evaluated as potential treatments for acute ischemic stroke, none have demonstrated a definitive treatment efficacy, which remains elusive. HT047 is an herbal extract of *Scutellaria baicalensis* and *Pueraria lobata*, both of which have been widely used to treat ischemic stroke in traditional Korean medicine. The aims of this trial are to investigate whether HT047 can improve neurologic status, particularly motor function, in acute ischemic stroke patients, and to determine the safety of HT047.

**Methods:**

A multicenter, double-blind, randomized, placebo-controlled, 3-arm parallel group, phase II trial will be conducted in patients who have had an acute ischemic stroke within the past 14 days. The participating patients must have a Fugl-Meyer assessment (FMA) motor score ≤55, with arm or leg weakness, and Korean version of the National Institutes of Health Stroke scale (K-NIHSS) score of ≥4 and ≤15. Seventy-eight participants will be randomized in a 1:1:1 ratio and given high-dose HT047 (750 mg 3 times a day), low-dose HT047 (500 mg 3 times a day), or a placebo for 12 weeks. The primary endpoint is the change in FMA motor score between baseline and week 12. Secondary endpoints are as follows: the change in FMA motor score at weeks 4 and 8 from baseline; the change in FMA motor score at weeks 4, 8, and 12 from baseline according to the timing of treatment initiation (either within 1 week, or 1–2 weeks), or according to the presence of prognostic risk factors (hypertension, diabetes, dyslipidemia, etc); the change in K-NIHSS and Korean versions of the modified Rankin scale (K-mRS) and the modified Barthel index at weeks 4 and 12 from baseline; and the proportion of subjects at week 12 with a K-NIHSS score of 0 to 2, or with K-mRS scores of 0, ≤1, and ≤2.

**Discussion:**

This study is a 1st-in-human trial of HT047 to explore the efficacy and safety in acute ischemic stroke patients. The results will provide the appropriate dosage and evidence of therapeutic benefit of HT047 for stroke recovery.

**Trial registration:**

ClinicalTrials.gov (NCT02828540) Registered July 11, 2016.

## 1 Introduction

Ischemic stroke remains a leading cause of death and serious long-term disability worldwide. Intravenous thrombolysis and endovascular treatment have proven to be the most effective treatments for acute ischemic stroke. However, because of the narrow time window available for these treatments, only a minority of patients receive these treatments, warranting the need for alternative therapeutic approaches.

Despite the failure of several clinical trials, neuroprotection remains a promising alternative or adjunct treatment of acute stroke.^[1]^ Recent advances have revealed that the induction of endogenous neurorestorative processes in the brain, including neurogenesis, synaptogenesis, vascular and axonal remodeling, and oligodendrogenesis aid in neurologic recovery during the subacute and chronic phases.^[2]^ Neurorestoration has increasingly become an important therapeutic approach, which offers a substantially longer therapeutic time window than the limited time window available for drugs with neuroprotective properties, because neurons in the ischemic penumbra only survive temporarily.^[1]^ To facilitate an optimal functional recovery after ischemic stroke, attempts have been recently made to develop drugs with both neuroprotective and neurorestorative properties.^[3–5]^ In this regard, many natural products have been reported to possess both of these properties as they contain a wide range of bioactive compounds.^[6]^

The HT047 is a multiherbal extract of the roots of *Pueraria lobata* and *Scutellaria baicalensis*. It was developed by screening more than 200 herbs used in traditional Korean medicine for the treatment of stroke, with the aim of promoting both neuroprotection and neurorestoration following ischemic stroke. *P lobata*, *S baicalensis*, and their major active compounds, which include puerarin, daidzin, and daidzein in *P lobata*, and baicalin, baicalein, and wogonin in *S baicalensis*, are well known for their neuroprotective effects against cerebral ischemia.^[7–10]^ Moreover, previous studies have shown that the major active compounds of HT047 (puerarin, daidzein, baicalin, baicalein, and wogonin) stimulate key restorative processes, including angiogenesis, neurogenesis, axonal sprouting, dendritic branching, and synaptogenesis.^[11–18]^ Based on these preclinical findings, we designed this study protocol to test the efficacy and safety of 12-week treatment with HT047 in acute ischemic stroke patients with motor deficits.

### 1.1 Objectives

The primary objective is to examine the efficacy of HT047 for the recovery of motor function in patients with acute ischemic stroke. The secondary objectives are to investigate whether HT047 enhances neurologic recovery and to evaluate the safety of HT047. The exploratory objective is to assess the efficacy of HT047 on cognitive function.

### 1.2 Design

This study is a phase 2, multicenter, double-blind, randomized, placebo-controlled, superiority trial with 3 parallel groups. Eligible participants will be randomly assigned to 1 of 3 groups: HT047 high- or low-dose, or placebo with a 1:1:1 allocation ratio, and will receive the investigational product for 12 weeks.

## 2 Methods

### 2.1 Study setting

The participants will be recruited from eight institutes in the Republic of Korea, including the Departments of Neurology at Kyung Hee University Hospital (Seoul), Kyung Hee University Hospital at Gangdong (Seoul), Hanyang University Hospital (Seoul), Hanyang University Guri Hospital (Gyeonggi-do), Hanyang University Myongji Hospital (Gyeonggi-do), Gachon University Gil Medical Center (Incheon), Hallym University Dongtan Sacred Heart Hospital (Gyeonggi-do), and Chosun University Hospital (Gwangju). Regular investigators’ meetings will be held to encourage enrollment and regular newsletters will be sent until the required sample size is reached. The 1st patient was enrolled in August 4, 2016.

### 2.2 Eligibility criteria

The inclusion and exclusion criteria are provided in Table 1. Briefly, patients are eligible if they have had an ischemic stroke confirmed by brain imaging within 14 days of screening, are aged 19 years or older, have arm or leg weakness with a Fugl-Meyer assessment (FMA) motor score ≤55 at screening, and have a Korean version of the National Institutes of Health Stroke scale (K-NIHSS) score ≥4 and ≤15 at screening.

**Table 1:**
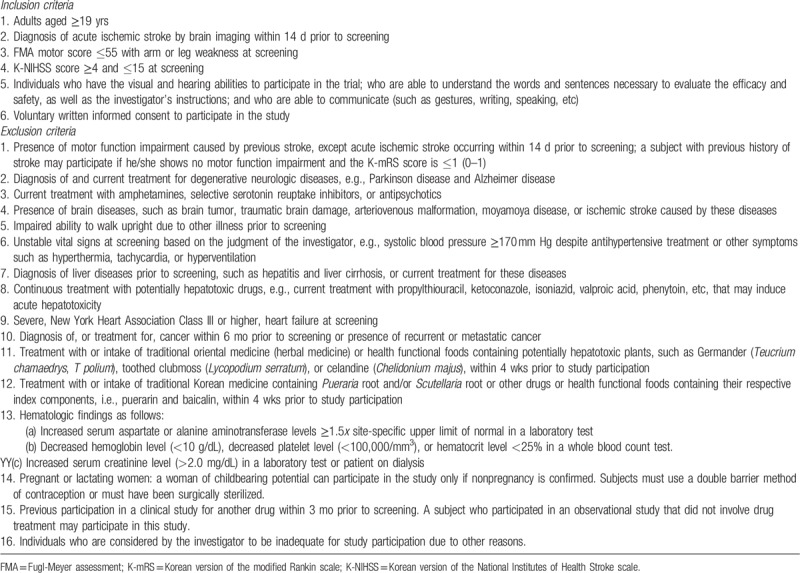
Inclusion and exclusion criteria.

### 2.3 Procedures

This study is designed to initiate treatment with high- or low-dose HT047 or placebo in subjects with acute ischemic stroke within 2 weeks of the onset of disease and evaluate neurologic and functional recoveries during 12 weeks of treatment in these subjects. The schedule for enrollment, interventions, and assessment of this study is shown in Table 2. The investigator will obtain written informed consent from subjects who voluntarily agreed to participate in the study before eligibility assessment. During the study treatment period, participants will visit the hospital at weeks 1, 4, 8, and 12. Since this is a 1st-in-human trial for HT047, subjects will have a study visit at week 1 to undergo laboratory tests, electrocardiogram, and chest X-ray, also, the subject's physical status will be examined before he/she is sent home. Neurologic function assessments will be carried out at subsequent visits. At week 12, the subject will again undergo laboratory tests, electrocardiogram, and chest X-ray and overall changes in the subject's status will be confirmed before all study procedures are ended.

**Table 2:**
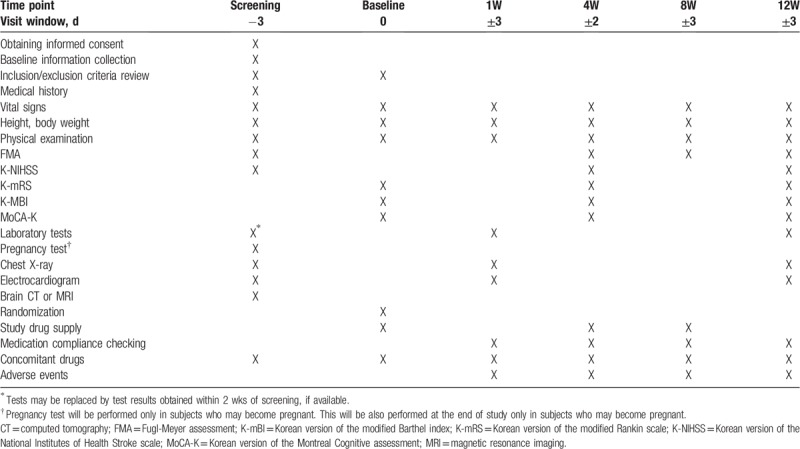
Study schedules.

### 2.4 Interventions

The HT047 will be produced by Bioland Co, Ltd, a bulk Good Manufacturing Practice-certified manufacturer (Osong, Chungcheongbuk-do, Republic of Korea). The dried roots of *P lobata* and *S baicalensis* will be mixed together in an 8.0:1.1 weight ratio and then be extracted twice with 30% ethanol in distilled water for 3 hours at 70°C. The liquid extract will be filtered, concentrated under 60°C, and vacuum dried to yield the extract, HT047. The study drug (a tablet containing 250 mg of HT047) and a placebo, identical in appearance to the study drug, will be provided by Kolmar Korea Inc (Sejong-si, Republic of Korea).

Participants will receive either the investigational product (HT047) or placebo 3 times a day for 12 weeks, starting from the morning after the baseline visit. Three tablets will be administered per dose as follows: 3 HT047 tablets for high-dose group (2250 mg/d), 2 HT047 tablets, and 1 placebo tablet for low-dose group (1500 mg), and three placebo tablets for placebo group. A one-month supply of the investigational products will be given to the subjects at baseline and weeks 4 and 8. At each visit, subjects will be asked to return the remaining investigational products for calculating their compliance and will be encouraged to adhere to the prescribed dosage regimen.

Physiotherapy sessions will be freely prescribed by the patient's attending physician, neurologist, or rehabilitation physician, according to medical opinion and subjects’ requests. Subjects will be prohibited from concomitant use of any medications which can induce hepatotoxicity, such as propylthiouracil, ketoconazole, isoniazid, valproate, and phenytoin; any medications containing *P lobata*, *S baicalensis*, or their major components (puerarin and baicalin, respectively); selective serotonin reuptake inhibitors; amphetamine; cholinesterase inhibitors, such as donepezil, rivastigmine, and galantamine; memantine; nootropics, such as choline alfoscerate, acetyl-l-carnitine, oxiracetam, and *Ginkgo biloba.*

The allocated intervention will be discontinued if a participant or a participant's legal representative wishes to discontinue study participation or if the investigator decides to discontinue due to medical or administrative reasons (adverse events, continuous protocol violations, etc).

### 2.5 Randomization and blinding

Eligible participants will be randomly assigned to one of the following 3 groups in a 1:1:1 ratio: high-dose (750 mg 3 times a day), low-dose (500 mg 3 times a day), and placebo groups. An independent statistician will generate a list of random numbers using a centralized stratified block randomization method. Randomization will be stratified according to the hospital, so that all hospitals have a 1:1:1 ratio. The study drug and placebo will be sealed in sequentially numbered identical bottles according to the allocation sequence. Each participant will be assigned an order number and will receive the investigational product or placebo in the corresponding prepacked bottles. Participants and all research personnel will be blinded to the assignment. The randomization code will be kept secure by an independent statistician and can only be broken in the case of an emergency. If a code break occurs, the investigator will report the reason and procedures and the administration of investigational drug will be discontinued.

### 2.6 Outcome measures and endpoints

The Korean version of the FMA motor score will be used to measure the primary endpoint.^[19]^ The motor domain of FMA ranges from a score of 0 (flaccid hemiplegia) to 100 (normal movement), with 66 points for the upper limb and 34 points for the lower limb. An educated physiotherapist will perform all motor assessments at baseline and weeks 4, 8, and 12. The K-NIHSS and Korean versions of the modified Rankin scale (K-mRS), the modified Barthel index (K-mBI), and the montreal cognitive assessment (MoCA-K) will be used at baseline and weeks 4 and 12.

The primary efficacy endpoint is the change in FMA motor score at week 12 from the baseline. The secondary efficacy endpoints are: the change in FMA motor score at weeks 4 and 8 from the baseline; the change in FMA motor score at weeks 4, 8, and 12 from baseline according to the timing of treatment initiation after the onset of stroke (either within 1 week or 1–2 weeks) or according to the presence of prognostic risk factors (hypertension, diabetes, dyslipidemia, etc); the change in K-NIHSS and K-mRS scores at weeks 4 and 12 from the baseline; the proportion of subjects with a K-NIHSS score of 0 to 2 at week 12; the proportion of subjects with a K-mRS score of 0, ≤1, and ≤2 at week 12; and the change in K-MBI score at weeks 4 and 12 from the baseline. Exploratory endpoint is the change in MoCA-K score at week 12 from baseline.

Safety endpoints are the adverse events, significant changes in vital signs and physical examination; and abnormal laboratory tests, electrocardiogram, and chest radiography results. Laboratory tests will include hematology, electrolyte, blood chemistry, lipid battery, blood coagulation, and urinalysis.

### 2.7 Sample size

The primary endpoint of the study is the change in FMA motor score at baseline from that after 12 weeks. A sample size of 22 in each group will be sufficient to detect a clinically important difference of 14.5 points on the FMA score, assuming a standard deviation (SD) of 19.2. This estimate is based on the findings of the fluoxetine in motor recovery of patients with acute ischemic stroke trial, which reported a difference of mean ± SD 90-day FMA score change between fluoxetine group (n = 57, 36.4 ± 21.3) and control group (n = 56, 21.9 ± 16.7).^[20]^

Because this HT047 trial is a phase 2 exploratory clinical trial for appropriate dose selection, we did not consider adjustment to control for type 1 errors resulting from the comparison between the 3 groups. However, in the comparison of primary efficacy outcome between the 3 groups, multiple comparisons will be performed to control for the experiment-wise error rate. We will use a Student *t* test of the difference between mean values, a power of 80%, and a significance level of 10%. With a 15% anticipated drop-out rate considered, we plan to enroll 26 patients per group (a total of 78 patients).

The formula for the sample size for comparison of 2 mean values is as follows:
 
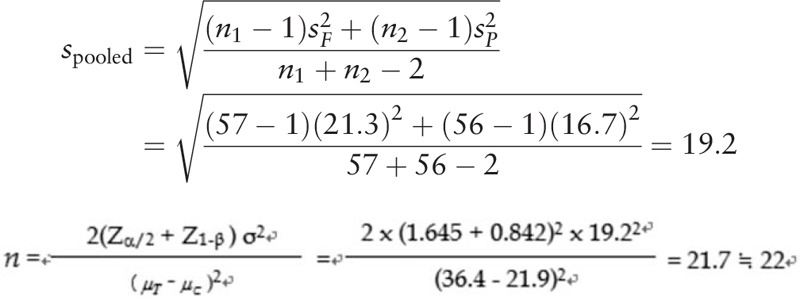


where *Z*_*α*/2_ is the normal distribution threshold of type 1 error (*α* = 0.10/2), *Z*_1-*β*_ is the normal distribution threshold of power (1 − β = 0.8), *μ*_*T*_ is the mean change of 12-week FMA score in HT047 group (high dose or low dose), *μ*_*C*_ is the mean change of 12-week FMA motor score in control group, and σ is the standard deviation.

### 2.8 Statistical analysis

Efficacy will be assessed in the full analysis set (FAS) and the per-protocol (PP) set. The FAS will include all subjects who meet the inclusion/exclusion criteria, provide voluntary informed consent to participate in the study, receive at least 1 dose of the investigational product after randomization, have FMA assessment, that is, the primary efficacy endpoint, at screening, and at least once at weeks 4, 8, or 12 during the follow-up period. The PP set will include all FAS subjects who do not have any major violations such as major protocol violations (excluding minor violations) and have a primary efficacy assessment per protocol. Subjects with an overall medication compliance of <70% will be excluded from the PP set. Safety will be assessed in the safety set, which will comprise of all subjects who receive at least 1 dose of the investigational product after randomization.

The primary efficacy endpoint will be analyzed both in the FAS and the PP sets. The final conclusions of the study will be made based on the FAS results, in principle. Mean, SD, median, and range (minimum, maximum) of FMA motor score before dosing and 12 weeks after dosing, as well as score change between the 2 time points, will be presented for the high- and low-dose HT047, and the placebo groups. To assess intergroup differences in the primary endpoint, that is, FMA change, analysis of covariance, with baseline FMA as a covariate, and Tukey multiple comparison test will be carried out. Dunnett multiple comparison, which would consider the placebo group as a comparator group, will not be used because this phase 2 study aims to evaluate adequate dosage and therefore, will compare the HT047 high-dose group with the HT047 low-dose group.

The secondary efficacy and exploratory endpoints will be also analyzed both in the FAS and the PP set, with the final conclusions to be made based on the PP set results. For each secondary efficacy and exploratory endpoint, mean, SD, median, and range (minimum, maximum) will be presented by the group for continuous data, whereas frequency and percentage will be provided by the group for categorical data. For the following secondary efficacy endpoints, generalized estimating equation analysis will be conducted for overall data or by subgroup, according to the relevant definition. For generalized estimating equation analysis, the following intergroup differences in FMA score change at each time point will be assessed by performing a contrast analysis, while a trend analysis will be conducted to characterize the trend in FMA change between time points by group: intergroup comparison of change in FMA motor score at weeks 4 and 8 from the baseline; intergroup comparison of FMA motor score change at weeks 4, 8, and 12 from the baseline according to the timing of treatment initiation after the onset of stroke (either within 1 week or 1–2 weeks), and according to the presence of prognostic risk factors (hypertension, diabetes, dyslipidemia, etc); intergroup comparison of change in K-NIHSS and K-mRS scores at weeks 4 and 12 from the baseline; and intergroup comparison of change in K-MBI score at weeks 4 and 12 from the baseline. For the following secondary efficacy endpoints, a Chi-squared test will be conducted, and if there is an intergroup difference in proportion, a Hochberg test will be carried out to compare the HT047 high-dose, HT047 low-dose, and placebo groups to control for multiplicity issues: intergroup comparison of the proportion of subjects with K-NIHSS score 0 to 2 at week 12; intergroup comparison of the proportion of subjects with K-mRS score 0 at week 12; intergroup comparison of the proportion of subjects with K-mRS score ≤1 at week 12; and intergroup comparison of the proportion of subjects with K-mRS score ≤2 at week 12. To assess intergroup difference in the exploratory endpoint, that is, change in MoCA-K score at week 12 from the baseline, analysis of covariance and Tukey multiple comparison test will be carried out.

A comprehensive statistical analysis plan will be finalized prior to database locking. Details of statistical analyses that are not presented in the protocol will be described in the statistical analysis plan. All statistical analyses will be performed at a 2-sided significance level of 10%. To handle the missing data, the last observation carried forward method will be used for FMA motor score, the primary outcome variable. If FMA motor score is measured only at screening, substitution for missing data will not be performed. Similarly, for the other outcome variables, missing data will not be substituted and included in the statistical analysis. Interim analysis will not be performed.

### 2.9 Data management and monitoring

All data will be entered and stored in a secure electronic data capture system. Site investigators will have direct access to the data set from their own sites and the sponsor will have access to the final trial data set. The identity of subjects will be concealed using a subject identification code in all study documents. Personal information obtained from this trial will be stored for up to 3 years after completion of the trial and will not be provided to anyone not involved in the study. Participating centers will be monitored by on-site visits of trained monitors in accordance with standard operating procedures during the entire course of the study. Source data verification will be performed during monitoring to improve the data quality. A Data Monitoring Committee will not be needed because this is a low-risk study and no interim analysis will occur.

### 2.10 Harms

All adverse events will be monitored and recorded, irrespective of causality. The investigators will evaluate the severity of symptoms and the causal relationship to the investigational products. All serious adverse events will be reported to the Institutional Review Board and sponsor within 24 hours. Participants will be compensated for medical expenses related to treatment of injury that is confirmed to be caused by the investigational products or procedure.

### 2.11 Ethics and dissemination

This protocol (ver 5.0, issue date July 12, 2017) has been approved by the ethics committees of Kyung Hee University Hospital (KHUH IRB 2016-03-303-037), Kyung Hee University Hospital at Gangdong (KHNMC 2016-02-016-011), Hanyang University Hospital (HYUH 2016-02-017-020), Hanyang University Guri Hospital (GURI 2016-03-003-020), Hanyang University Myongji Hospital (MJH 2016-01-014-025), Gachon University Gil Medical Center (GAIRB2016-102), Hallym University Dongtan Sacred Heart Hospital (HDT 2017-03-194-001), and Chosun University Hospital (CHOSUN 2017-03-010-002), and registered with ClinicalTrials.gov (NCT02828540, July 11, 2016). Written informed consent will be obtained from all the patients based on local ethics committee recommendations. A model consent form is also available from the corresponding author on reasonable request. This study is being conducted in accordance with the Declaration of Helsinki and the Korean Good Clinical Practice guidelines. The full protocol and participant-level data sets generated and/or analyzed during the present study are not publicly available. The results of this study will be published in a peer-reviewed journal

## 3 Discussion

Although neuroprotective agents have been tested for efficacy in several preclinical and clinical settings, none have been investigated in large clinical trials for ischemic stroke.^[21]^ Recent studies have assessed the efficacy of several agents, acting through various mechanisms, in patients who underwent thrombolytic treatment.^[1,22]^ Though oriental herbal medicines have been used to treat ischemic stroke for thousands of years, and even to this day, in Asian countries including Korea, China, and Japan, their efficacy remains unclear due to the poor quality of clinical trials. Consequently, clinical practice guidelines do not recommend any neuroprotective agents and herbal products to treat acute stroke in patients.

Certain herbal products have shown neuroprotective effects in clinical trials and have successfully improved poststroke recovery.^[23,24]^ However, these trials lack a randomized, double-blind, placebo-controlled design, which is important to investigate the efficacy of therapeutic interventions. Therefore, there is an unmet need to determine the efficacy and safety of herbal medicines through well-designed studies. Because the active compounds of *P lobata* and *S baicalensis* have been previously shown to have neuroprotective and neurorestorative effects,^[7–18]^ we designed this study protocol to investigate the efficacy and safety of HT047.

The FMA motor score was the primary endpoint to assess motor function. FMA is a quantitative assessment tool developed by Fugl-Meyer et al in 1975 to measure the functional restoration of stroke patients^[19]^ and consists of 5 domains (motor function, sensory function, balance, joint range of motor, and joint pain) with a total score of 226 points with excellent inter- and intrarater reliability and good validity.^[20,25]^ In the FMA, motor function scale has been mostly used, and we will only use the motor function scale in this trial. The evaluation of motor function involves the evaluation of the upper and lower extremities, and of synergy, with the advantage that both the upper and lower limbs can be evaluated simultaneously without any special equipment. The evaluation takes approximately 30 to 45 minutes. In this clinical trial, a specialist in the administration center has conducted offline training for researchers at individual institutions. In addition, we also use common scales including K-NIHSS, K-mRS, K-MBI, and MoCA-K for the secondary endpoint assessment.

This study protocol is not without limitations. First, this is a phase 2 pilot study comprising a relatively small sample size of patients. However, based on the results of this study, a subsequent phase 3 trial will be planned. Second, since our study will enroll patients whose stroke symptoms occurred within the previous 14 days, most patients will be in the subacute stage after stroke. Third, the study's drug formulation is a tablet that will be prescribed 3 times a day; patients with tube feeding due to dysphagia will be excluded. Another limitation is that evaluation of motor function is focused on the upper limb than the lower limb, and it lacks evaluation of fine movement. Further, FMA can also be prone to the ceiling effect. Nevertheless, there are several strengths of this protocol; it has a multicenter, prospective, and double-blind randomized design. In addition, we will investigate high and low doses of HT047, allowing for a stratified analysis for efficacy and safety. Furthermore, the FMA motor scale can provide detailed information concerning poststroke motor outcomes.

In conclusion, this study is a 1st-in-human trial of HT047 in acute ischemic patients. Our results will provide evidence on the efficacy and the safety of HT047 as a treatment to improve poststroke motor function and the appropriate dosage.

## Acknowledgment

The authors thank all the patients and therapists who supported the study in each of the 8 involved institutions for their collaboration and contribution to this study.

## Author contributions

**Conceptualization:** Hocheol Kim, Dae-il Chang

**Methodology:** Sung Hyuk Heo, Jungbin Song, Bum Joon Kim, Hocheol Kim, Dae-il Chang

**Project administration:** Hocheol Kim, Dae-il Chang

**Supervision:** Hocheol Kim, Dae-il Chang

**Writing – original draft:** Sung Hyuk Heo, Jungbin Song

**Writing – review & editing:** Bum Joon Kim, Hocheol Kim, Dae-il Chang
